# Discrimination between human normal renal tissue and renal cell carcinoma by dielectric properties using *in-vitro* BIA

**DOI:** 10.3389/fphys.2023.1121599

**Published:** 2023-03-17

**Authors:** Hang Wang, Xuetao Shi, Xinsheng Cao, Xiuzhen Dong, Lin Yang

**Affiliations:** ^1^ Department of Aerospace Medicine, Fourth Military Medical University, Xi’an, China; ^2^ Department of Biomedical Engineering, Fourth Military Medical University, Xi’an, China

**Keywords:** dielectric properties, characteristic parameters, renal cell carcinoma, distinguishing coefficient, human normal kidney

## Abstract

Renal cell carcinoma (RCC) poses a serious threat to human health, which urgently requires a method that can quickly distinguish between human normal renal tissue (NRT) and RCC for the purpose of accurate detection in clinical practice. The significant difference in cell morphology between NRT and RCC tissue underlies the great potential of the bioelectrical impedance analysis (BIA) to distinguish two types of human tissues. The study aims to achieve such discrimination through comparison of their dielectric properties within the frequency range from 10 Hz to 100 MHz. The dielectric properties of 69 cases of human normal and cancer renal tissue were measured 15 min after tissue isolation in a strictly controlled environment (37°C, 90% humidity). In addition to the impedance parameters (resistivity, conductivity and relative permittivity), the characteristic parameters extracted from the Cole curve were also compared between NRT and RCC. Furthermore, a novel index, distinguishing coefficient (DC), was used to obtain the optimal frequency for discrimination between NRT and RCC. In terms of impedance parameters, the RCC conductivity at low frequencies (<1 kHz) was about 1.4 times as large as that of NRT, and its relative permittivity was also significantly higher (*p* < 0.05). In terms of characteristic parameters, two characteristic frequencies (14.1 ± 1.1 kHz and 1.16 ± 0.13 MHz) were found for NRT while only one for RCC (0.60 ± 0.05 MHz). A significant difference of low-frequency resistance (R_0_) between RCC and NRT was also observed (*p* < 0.05). As for the new index DC, relative permittivity DCs below 100 Hz and at around 14 kHz were both greater than 1. These findings further confirm the feasibility of discrimination between RCC and NRT and also provide data in favor of further clinical study of BIA to detect the surgical margins.

## 1 Introduction

In recent years, the incidence of renal tumor has gradually increased, which poses a serious threat to human health (Makino et al., 2022). Renal cancer accounts for about 2.4% of all malignancies in adults, with more than 400,000 new cases diagnosed and about 180,000 deaths worldwide in 2020 according to GLOBOCAN data (Sung et al., 2021). An epidemiological survey reveals that the incidence of renal cancer in common tumors has been stable in the top eight in United Kingdom and United States and accounts for 4% of all new cancer cases (Rai et al., 2022; Siegel et al., 2022). The incidence of renal tumors is growing steadily every year (Klatte et al., 2015) and approximately 90% of primary renal tumors are renal cell carcinoma (RCC) (Goodarzi et al., 2018).

The widespread use of contemporary imaging techniques has made the detection of small incidental renal tumors possible (Inagaki et al., 2004). Diagnosed at an early stage, RCC is often dealt with partial nephrectomy, which is an accepted surgical procedure for localized RCC (Klatte et al., 2015). The treatments of RCC include surgery, radio frequency ablation and cryoablation therapy, in all of which a layer of normal renal tissue (NRT) is often removed with RCC tissue in order to avoid positive surgical margins. The thickness of NRT mainly relies on the surgical experience of the doctor and a smaller surgical margin is known to be beneficial for the reduction of postoperative injuries (Lam et al., 2008). Therefore, a detection method that can quickly distinguish between NRT and RCC is highly required in clinical practice to accurately detect the surgical margins.

Bioelectrical impedance is one of the essential biophysical properties of biological tissue, which is determined by the tissue microstructure. Each tissue has specific dielectric properties, such as conductivity, permittivity, resistivity and so on. Numerous studies have demonstrated that tissue dielectric properties are highly correlated to cell arrangement and microstructure that reflect physiological or pathological information of the tissue. Also, the measurement of bioelectrical impedance is very easy to carry out, in which safe currents are injected into the targeted human body and the boundary voltages induced by body’s internal tissue are measured through the surface electrodes. So far, BIA has been widely used in biomedical applications, such as stroke detection, cancer detection and ventilation monitoring (Brown et al., 2000). Because significant difference exists in cell morphology between NRT and RCC tissue, BIA has great potential to distinguish between NRT and RCC in theory.

Up to now, several studies have investigated the dielectric properties of NRT and RCC. Takeshi *et al* (Inagaki et al., 2004) measured the capacitance of *ex vivo* human kidney tissues freshly obtained after surgical excision at the frequency of 1 MHz, and found the ratio of tumor-to-normal tissue dielectric permittivity could be up to about 1.4 for cell carcinoma. However, the *ex vivo* time was not strictly controlled and recorded, which resulted in a large standard deviation in the measurement results. Yun *et al* (Yun et al., 2016) found a significant difference in dielectric spectroscopy between human normal and cancer renal tissue by using a micro electrical impedance spectroscopy-on-a-needle (two-electrode strategy) at frequencies from 100 Hz to 1 MHz. But they pointed out that non-negligible needle distortions might occur when the boundaries between the tissues were unclear, which led to an inaccurate extraction of dielectric properties of tissue (Kim et al., 2019). Additionally, similar results from animal tissue were also obtained (TV et al., 2020). These previous studies showed the difference in the dielectric properties between NRT and RCC, but the measurement results could not be directly compared for clinical use. First, the measurement conditions were not strictly controlled, such as temperature, humidity and *ex vivo* time. Second, the measurement parameters were inconsistent, such as frequency range and strategy of electrode use. Third, the tissue samples were not all from the human body. In conclusion, to our knowledge, no study has carried out a comprehensive measurement as well as analysis of the dielectric properties of human NRT and RCC in a wide frequency range within as short as possible an *ex vivo* time.

In this study, the dielectric properties of both NRT and RCC from human body were measured from 10 Hz to 100 MHz in a strictly controlled environment (37 °C, 90% humidity) within 15 min after the tissue was isolated. In addition, the dielectric properties of NRT and RCC were comprehensively compared to obtain the quantitative indices to differentiate between NRT and RCC.

## 2 Methods and materials

### 2.1 Ethical statement and sample source

This study was approved by the Medical Ethics Committee of the Fourth Military Medical University [Approval No. FMMU-E-III-001(1)]. The experiment was conducted in accordance with the Declaration of Helsinki 1975, as revised in 2000.

A total of 69 renal tissue samples were obtained from 41 patients who underwent nephrectomy for renal tumor in the general surgery operating room of Xijing Hospital. All tissue samples were stained for pathological HE and finally confirmed for cancer type by the Pathology Department of Xijing Hospital. The tumor types of 41 patients included renal clear cell carcinoma (34 cases), papillary carcinoma (4 cases), and chromophore cell carcinoma (3 cases), which all belong to RCC. Another 28 cases of normal renal parenchyma were taken from the tissues adjacent to tumor. Because the renal clear cell carcinoma is the most common type of RCC (Hsieh et al., 2017), this study mainly focuses on the comparison of the dielectric properties of NRT and renal clear cell carcinoma.

### 2.2 Measurement of dielectric properties

**FIGURE 1:**
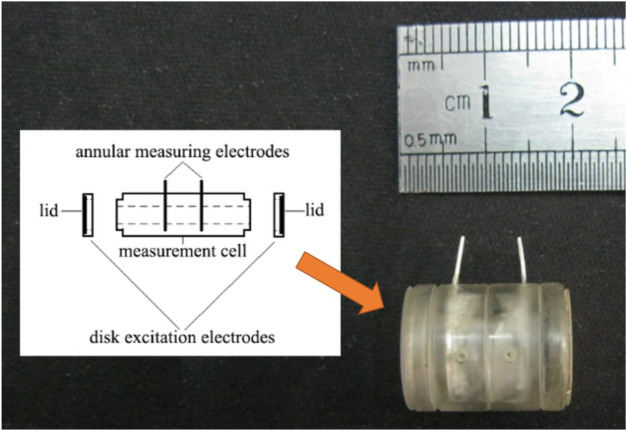
Measurement cell with four electrodes.

During operation, a tissue sample was immediately sent to the infant incubator (Daiwei, Ningbo, China) following excision. Then the tissue was clipped into a measurement cell after being trimmed to ensure the measurement accuracy. The measurement cell was cylindrical (Figure 1), whose structural parameters were described in our previous studies (Wang et al., 2021). The two electrodes embedded at the lid of the cell served as excitation electrodes, and the other two electrodes embedded in the inner wall of the cell functioned as measuring electrodes. The whole measurement process was performed in the infant incubator to maintain a constant environment (37°C, 90% humidity). Additionally, in order to shorten the *ex vivo* time as much as possible, all measurements were completed within 15 min after the tissue was isolated.

The measurement of dielectric properties was implemented by using the measurement platform developed by our group, which consisted of two impedance analyzers (Wang et al., 2021). On one hand, the Solartron1260 impedance analyzer (Schlumberger, United Kingdom) with a Solartron 1294 biological impedance interface was employed to measure the dielectric properties from 10Hz to 1 MHz with the four-electrode method, which was used to eliminate the polarization effect of the electrode and contact impedance. On the other hand, thee Agilent 4294A impedance analyzer (Agilent Technologies, United States) was utilized to measure the dielectric properties from 10kHz to 100 MHz with the two-electrode method, which was applied to minimize the adverse effect of stray capacitance and inductance from the measurement cell and wire on the recorded results. A 0.5 mA RMS signal was adopted across two exciting electrodes with logarithmically sweeping frequency with a manner of 10 points per tenfold frequency for both the impedance analyzers. Moreover, before measurement of human tissue, both the impedance analyzers were calibrated by using a 0.03 mol/L saline solution. The reliability of our measurement platform has been verified in our previous studies (Wang et al., 2015).

### 2.3 Analysis of dielectric properties

#### 2.3.1 Impedance parameters

The complex admittance *Y* of human tissue can be denoted by:

$$Y=G+j\omega C=\left(\frac{S}{L}\right)\left(\sigma +j\omega \varepsilon_{0}\varepsilon_{r}\right)$$ (1)

where*ω*is the angular frequency; $\varepsilon_{0}$ is the vacuum dielectric constant; *S* is the cross-sectional area of the measured sample; *L* is the effective length of the measured sample; σ is conductivity; and $\varepsilon_{r}$ is relative permittivity.

With the Solartron 1260 and the Agilent 4294A systems, the tissue impedance was obtained, which consisted of the real part *Re* and imaginary part *Im*. Thus, *Y* could also be written as:

$$Y=\frac{1}{Z}=\frac{1}{Re+jIm}=\frac{Re-jIm}{{Re}^{2}+{Im}^{2}}=\frac{Re}{{Re}^{2}+{Im}^{2}}-j∙\frac{Im}{{Re}^{2}+{Im}^{2}}$$ (2)

where j is the unit imaginary number.

According to the measurement principle, the conductivity σ and relative permittivity $\varepsilon_{r}$ could be calculated as follows:

$$\sigma =\frac{L}{S}Re\;Y=\frac{L∙Re}{S\left({Re}^{2}+{Im}^{2}\right)}$$ (3)

$$\varepsilon_{r}=\frac{L}{S\omega \varepsilon_{0}}Im\;Y=-\frac{L∙Im}{S\omega \varepsilon_{0}\left({Re}^{2}+{Im}^{2}\right)}$$ (4)

As a result, the real part $\rho_{re}$ and imaginary part $\rho_{im}$ of the complex resistivity (*ρ*) of the measured tissue could be denoted as follows, in which conductivity, relative permittivity and the size of the measurement sample are related:

$$\rho_{re}=\frac{\sigma }{\sigma^{2}+{\left(\omega \varepsilon_{r}\varepsilon_{0}\right)}^{2}}∙\frac{L}{S}$$ (5)

$$\rho_{im}=-\frac{\omega \varepsilon_{r}\varepsilon_{0}}{\sigma^{2}+{\left(\omega \varepsilon_{r}\varepsilon_{0}\right)}^{2}}∙\frac{L}{S}$$ (6)

#### 2.3.2 Characteristic parameters

In addition to analyzing the resistivity, conductivity and relative permittivity, the characteristic parameters of NRT and RCC from the Cole curve were also extracted. The dielectric properties of the tissue could be denoted by the Cole formula:

$$Z\left(f\right)=R_{\infty }+\frac{R_{0}-R_{\infty }}{1+{\left(j\frac{f}{f_{c}}\right)}^{\alpha }}$$ (7)

where *R*_*0*_ is the impedance under direct current; *R*_*∞*_ is the impedance at infinite frequency; *α* is the dispersion parameter ( 1>α≥0 ); *f*_*c*_ is the characteristic frequency of the tissue (Cole, 1932). Biological tissue has different dispersion intervals resulting from different occurrence mechanisms (Schwan, 1993). To denote multiple dispersion intervals, the Cole formula was expanded as the following form:

$$Z\left(f\right)=R_{\infty }+\sum_{i=1}^{n}\frac{∆R_{i}}{1+{\left(j\frac{f}{f_{ci}}\right)}^{\alpha_{i}}}$$ (8)

where *i* indicates the *i*th dispersion interval; *ΔR*_*i*_ is the impedance increment in the *i*th dispersion interval. All characteristic parameters were used to distinguish NRT from RCC tissue.

#### 2.3.3 Optimal frequency for discrimination between RCC and NRT

In order to remove the magnitude difference of dielectric parameter in the whole frequency range and to reflect the reproducibility of the experimental results, a new index, distinguishing coefficient (DC), was used to obtain the optimal frequency. DC for two different types of tissue was defined by:

$$DC=\frac{M_{c}-M_{n}}{D_{c}+D_{n}}$$ (9)

where M, D, c, and n denote the mean value, standard deviation, cancer tissue, and normal tissue, respectively.

In this study, DC was respectively calculated for four impedance parameters including σ , $\varepsilon_{r}$ , $\rho_{re}$ and $\rho_{im}$ , which are frequency-dependent.

### 2.4 Statistical analysis

In this study, SPSS 23.0 (IBM Software, Armonk, NY) was employed for statistical analysis. The impedance parameters ( σ ; $\varepsilon_{r}$ ; $\rho_{re}$ ; $\rho_{im}$ ) and the characteristic parameters (*R*_*0*_, *R*_*∞*_, *α* and *f*_*c*_) were compared at different frequencies between NRT and RCC with independent sample *t*-test analysis. *p* < 0.05 was deemed statistically significant. To evaluate the ability of impedance parameters for discrimination between NRT and RCC, the receiver operator characteristics (ROC) analysis was employed to calculate the sensitivity, specificity, and area under the ROC curve (AUC). In the ROC analysis, the four parameters ( σ ; $\varepsilon_{r}$ ; $\rho_{re}$ ; $\rho_{im}$ ) at the frequency with largest DCs were selected to discriminate between NRT and RCC.

## 3 Results

### 3.1 Difference in impedance parameters between NRT and RCC

**FIGURE 2:**
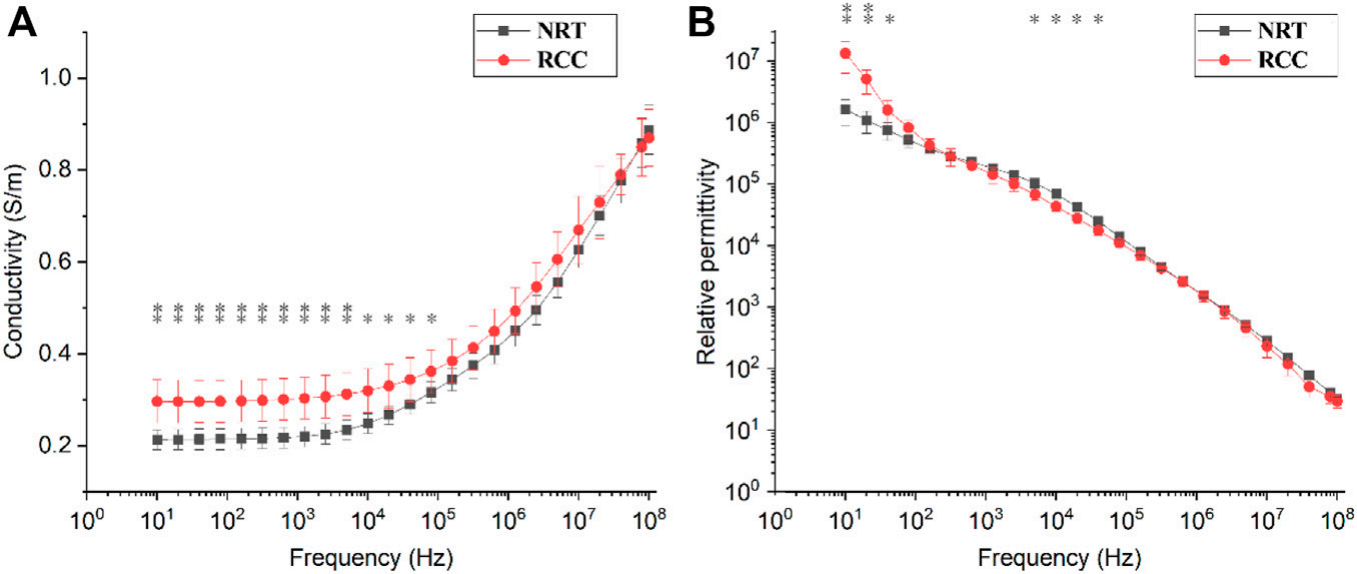
Changes in **(A)** conductivity and **(B)** relative permittivity of normal renal tissue (NRT) and renal cell carcinoma (RCC) with frequency. *p < 0.05, **p < 0.01.

Figure 2 shows the changes in conductivity and relative permittivity of NRT and RCC tissue with frequency. The NRT conductivity changed slightly below 5kHz, as did the RCC conductivity. But across this frequency range, there were significant differences in the conductivity between NRT and RCC (*p* < 0.05, Figure 2A). Above 5 kHz, the conductivity of both NRT and RCC increased rapidly with frequency. In contrast, the relative permittivity of both NRT and RCC had a fast and monotonic decrease with frequency across the whole frequency. However, the relative permittivity of RCC was significantly higher than that of NRT below 100 Hz (*p* < 0.05) whereas the relative permittivity of NRT was significantly higher than that of RCC from 5 kHz to 40 kHz (*p* < 0.05, Figure 2B).

**FIGURE 3:**
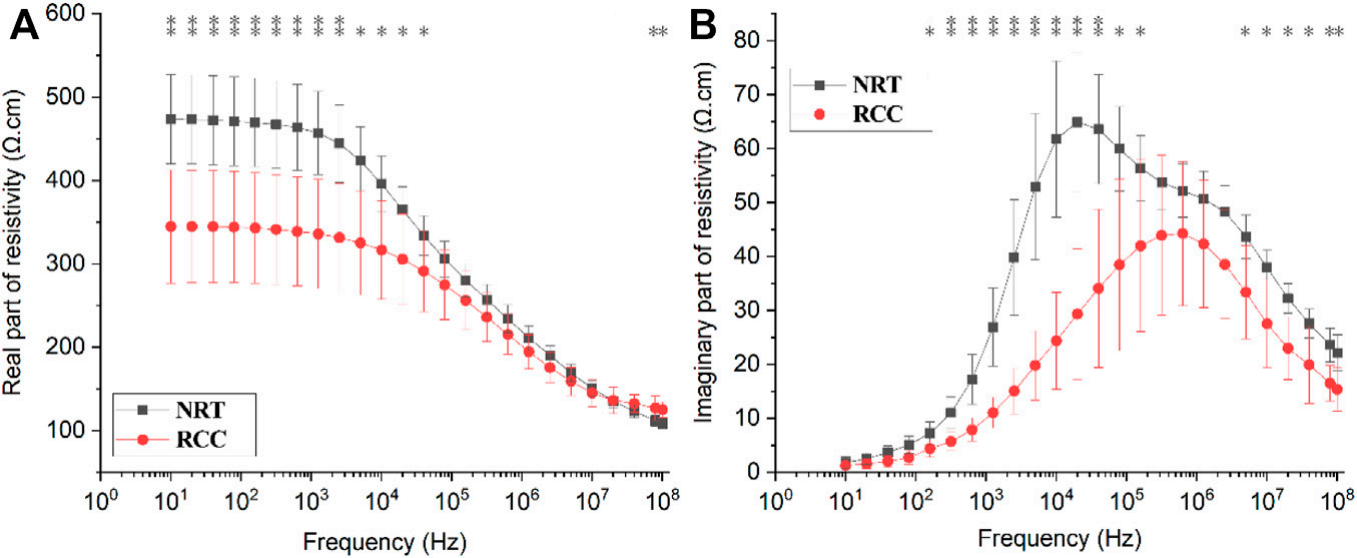
Changes in the **(A)** real part (ρre) and **(B)** imaginary part (ρim) of resistivity of normal renal tissue (NRT) and renal cell carcinoma (RCC) with frequency. *p < 0.05, **p < 0.01.

Figure 3 shows the changes in $\rho_{re}$ and $\rho_{im}$ of NRT and RCC tissue with frequency. While $\rho_{re}$ of NRT and RCC decreased with frequency, $\rho_{re}$ of NRT was significantly greater than that of RCC below 5 kHz and above 90 MHz (*p* < 0.05). Over the frequency, $\rho_{im}$ of NRT increased rapidly to the first peak (65.2 ± 13.6 Ω cm) at 14 kHz before starting to decrease, and continue to fall following a second peak (50.4 ± 5.1 Ω cm) at 1.2 MHz. For $\rho_{im}$ of RCC, it had a similar trend with a peak (44.2 ± 14.3 Ω cm) at 600 kHz. $\rho_{im}$ of NRT was significantly larger than that of RCC within 100 Hz–200 kHz and above 5 MHz.

### 3.2 Difference in characteristic parameters between NRT and RCC

**FIGURE 4:**
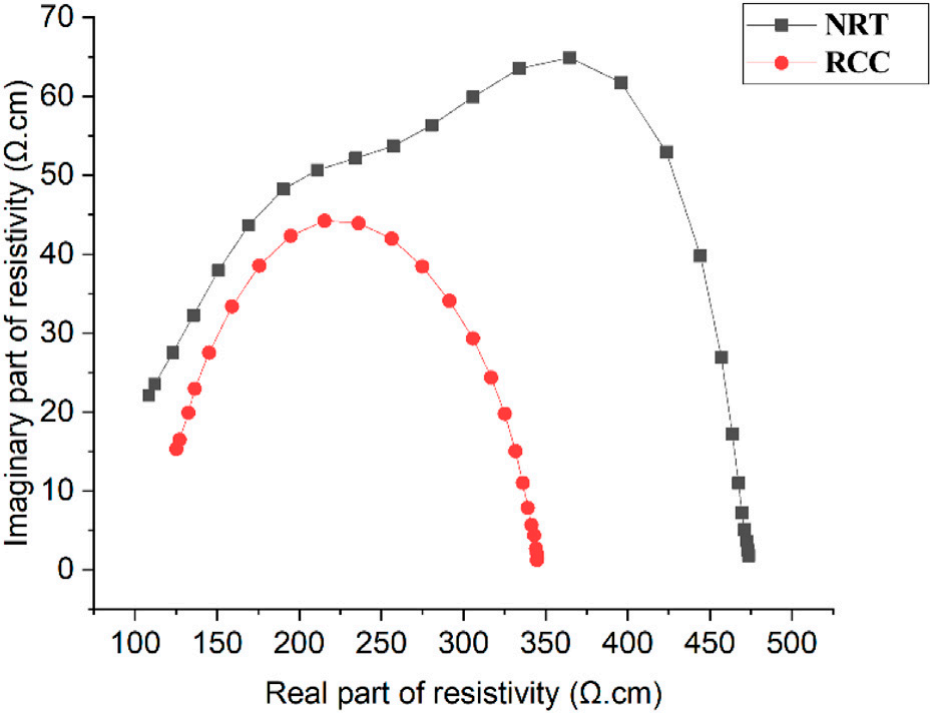
Cole–Cole curves of normal renal tissue (NRT) and renal cell carcinoma (RCC).

Figure 4 shows the Cole-Cole curves for NRT and RCC by using both mean of $\rho_{re}$ and $\rho_{im}$ at each frequency. Obviously, there were two dispersions for NRT while there was only one dispersion for RCC. The dispersion position for RCC approximated that of the second dispersion for NRT. Table 1 shows the characteristic parameters of NRT and RCC. Significant differences between NRT and RCC were found in terms of R_0_ (*p* < 0.01), f_c2_ (*p* < 0.001) and R_∞_ (*p* < 0.01).

**TABLE 1:** The characteristic parameters of normal renal tissue and renal cell carcinoma.

	R_0_(Ω)	f_c1_ (kHz)	α_1_	ΔR_1_(Ω)	f_c2_ (MHz)	α_2_	ΔR_2_(Ω)	R_∞_(Ω)
NRT	480 ± 21	14.1 ± 1.1	0.77 ± 0.03	124 ± 12	1.16 ± 0.13	0.42 ± 0.03	279 ± 13	76 ± 5
RCC	346 ± 55**	---	---	---	0.60 ± 0.05***	0.45 ± 0.04	249 ± 22	97 ± 12**

*p < 0.05; **p < 0.01; ***p < 0.001.

### 3.3 Optimal frequency to distinguish between RCC and NRT

**FIGURE 5:**
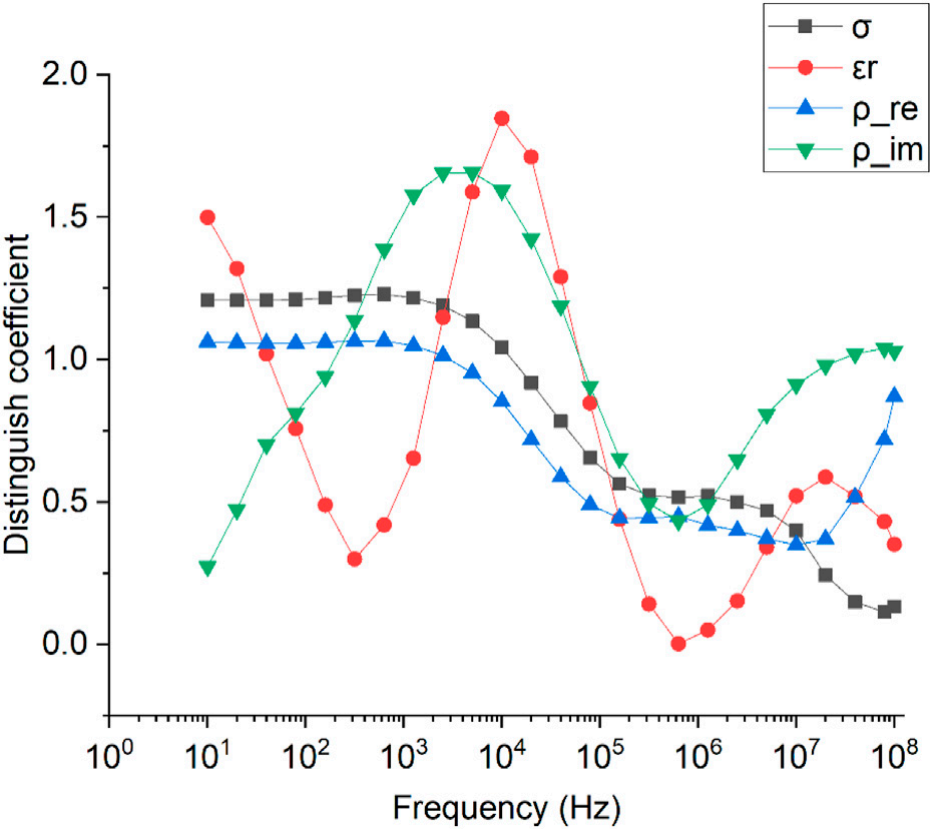
Changes in distinguishing coefficients of σ, $\varepsilon_{r}$ , $\rho_{re}$ and $\rho_{im}$ with frequency for normal renal tissue (NRT) and renal cell carcinoma (RCC).

Figure 5 shows the DC changes of σ , $\varepsilon_{r}$ , $\rho_{re}$ and $\rho_{im}$ with frequency for normal renal tissue (NRT) and renal cell carcinoma (RCC). Comparatively, $\varepsilon_{r}$ had the largest DC around 14 kHz, indicating that $\varepsilon_{r}$ around 14 kHz might have the optimal discrimination capability. While $\rho_{im}$ had the largest DC around 10 kHz. DC values of σ and $\rho_{re}$ under 1 KHz have hardly changed, and the largest DC of them appeared at around 633 Hz. The DC changes of σ and $\rho_{re}$ are similar below 10 MHz.

**FIGURE 6:**
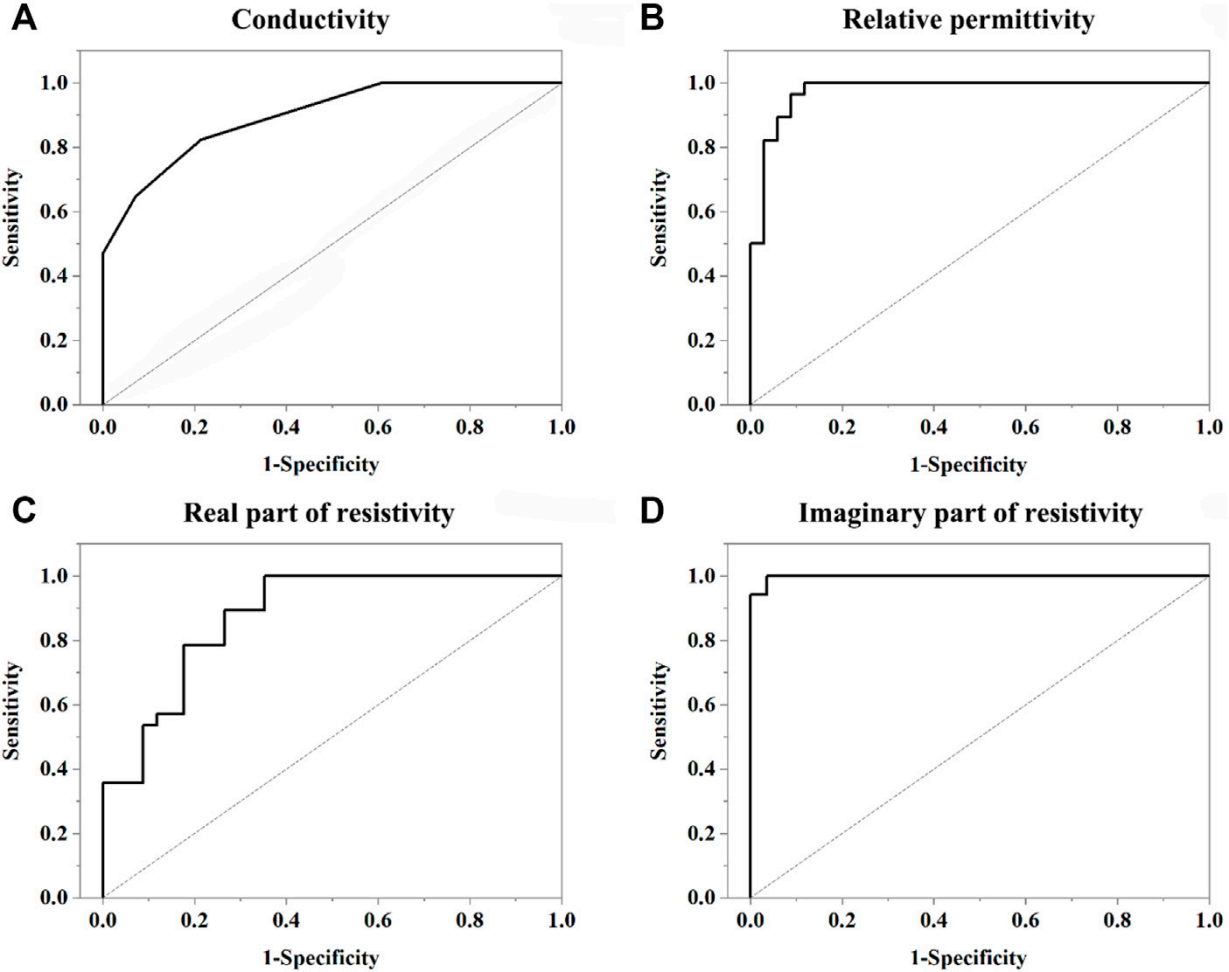
Receiver operating characteristic (ROC) curve of dielectric parameters (σ, $\varepsilon_{r}$ ; $\rho_{re}$ ; $\rho_{im}$ ) for ability to predict RCC. **(A)** Conductivity at 633 Hz. **(B)** Relative permittivity at 14 kHz. **(C)** Real part of resistivity at 633 Hz. **(D)** Imaginary part of resistivity at 10 kHz. The areas under the ROC curve (AUC) of σ, $\varepsilon_{r}$ , $\rho_{re}$ and $\rho_{im}$ were 0.896, 0.975, 0.876, and 0.997 for RCC prediction, respectively. The sensitivity and specificity ranged from 0.823 to 1 and from 0.735 to 0.964, respectively.

ROC analysis showed that the AUCs of σ, $\varepsilon_{r}$ , $\rho_{re}$ and $\rho_{im}$ were 0.896, 0.975, 0.876, and 0.997 for RCC prediction, respectively. The sensitivity and specificity ranged from 0.823 to 1 and from 0.735 to 0.964, respectively (Figure 6; Table 2).

**TABLE 2:** Area under the receiver operator characteristic curves for ability to predict RCC for dielectric parameters.

	σ	$\varepsilon_{r}$	$\rho_{re}$	$\rho_{im}$
AUC	0.896	0.975	0.876	0.997
Cut-off value	0.225	53,570.03	411.00	34.69
J-youden	0.609	0.876	0.628	0.964
Sensitivity	0.823	0.964	0.892	1
Specificity	0.785	0.911	0.735	0.964

AUC, area under receiver operator characteristic curve.

## 4 Discussion

In this study, the dielectric properties of human NRT and RCC were measured within a wide frequency range from 10 Hz to 100 MHz in a strictly controlled environment (37°C, 90% humidity) within 15 min after the tissue was isolated, and all dielectric parameters were comprehensively compared.

Previous studies showed that the dielectric properties of biological tissue were closely related to the microscopic state of the tissue, which were primarily influenced by temperature, humidity and, in particular, *ex vivo* time (Wang et al., 2021; Li et al., 2022). With the prolongation of *ex vivo* time, the lysosome membrane broke and various hydrolases were released, which hydrolyzed the cell components, and even resulted in the collapse of cell morphology and structure (Wang et al., 2015). These changes of the microscopic state of tissue further caused the variation of ionic content and mobility, which directly altered the flow path of the current. Thus, the dielectric properties of tissue gradually changed with *ex vivo* time. This may be the reason to explain the large difference in the dielectric properties of the same tissue measured by different research groups (Gabriel et al., 1996; TV et al., 2020). In this study, to reduce the effect of *ex vivo* time on tissue dielectric properties, all measurements were carried out within 15 min after the tissue was isolated, and temperature and humidity were maintained at levels close to an *in vivo* state.

Takeshi *et al* (Inagaki et al., 2004) found that tissue capacitance measurement was useful to distinguish between renal tumor tissue and normal kidney parenchyma in *ex vivo* tissue. However, they focused mainly on the capacitance characteristics at 1 MHz and did not analyze the dielectric properties over a wide frequency range, which contains more valuable information to characterize tissue state. Yun *et al* (Yun et al., 2016) specially designed a micro electrical needle to measure the dielectric properties of normal renal and cancer tissue from 100 Hz to 1 MHz with the two-electrode strategy. Nevertheless, the measured dielectric properties might be severely affected by electrode polarization because electrode-tissue contact impedance was largely greater than tissue impedance at the low frequencies (below 1 kHz) (Gabriel et al., 2009). In this study, the dielectric properties of NRT and RCC were measured within a wide frequency range from 10 Hz to 100 MHz by combining the four-electrode and two-electrode method, and the dielectric parameters were further compared, including impedance parameters, characteristic parameters and optimal frequency to distinguish between RCC and NRT.

Originated from the renal epithelium, RCC can be divided into 10 subtypes, including clear cell carcinoma, papillary renal cell carcinoma, chromophobe cell carcinoma and collecting duct carcinoma. Of all the subtypes, clear cell RCC is the most common and accounts for most cancer-related deaths (Rini et al., 2009) (Lobo et al., 2022), which is also the chief subtype of the measured RCC samples in this study determined by pathological results. Because clear cell RCC arises from renal parenchyma, renal parenchyma surrounding the RCC was excised to be measured for dielectric properties so that a direct comparison could be made in this study. We found that the conductivity of RCC at low frequencies (<1 kHz) was about 1.4 times as large as that of NRT, and the relative permittivity was also significantly greater than that of NRT (Figure 2). This may be related to the significant difference in the histological structure between RCC and NRT. Compared with normal tissue, the tumor cells are often enlarged with broad transparent cytoplasm and also rich in glycogen and lipids (Iwamoto et al., 2018). Such changes in the histological structure enhance the ability of cells to conduct electricity and store charges.

Biological tissue usually exhibit different dispersions as frequency changes, including α-dispersion (from 10 Hz to ∼10^3^ Hz), β-dispersion (from 10^3^ to <10^7^ Hz), δ-dispersion (<10^10^ Hz) and γ-dispersion (>10^10^ Hz) (Schwan, 1957). As shown in Figure 2B, the relative permittivity of NRT and RCC had a strong frequency dependence within the low frequency range from 10 Hz to 100 Hz, which might belong to α-dispersion produced by ionic diffusion at the site of the cellular membrane at low frequencies (Schafer et al., 1999) (Monai et al., 2012). Correspondingly, a large peak (about 1.5) in DC for $\varepsilon_{r}$ was found around 10 Hz (Figure 5), indicating that the information on $\varepsilon_{r}$ around 10 Hz could be useful to distinguish between NRT and RCC. Another dielectric parameter with significant difference between NRT and RCC was the characteristic frequency (*f*_*c*_). There were two characteristic frequencies for NRT (14.1 ± 1.1 kHz and 1.16 ± 0.13 MHz) while only one for RCC (0.60 ± 0.05 MHz). This phenomenon was also observed in the dielectric properties of human cancerous and normal lung tissue (Wang et al., 2014). These changes of dielectric properties might belong to β-dispersion, which principally attributed to the polarization of cellular membranes that block the ion flow between intracellular and extracellular media [20]. Additionally, a significant difference in the low-frequency resistance (R_0_) and the infinite-frequency resistance (R_∞_) between NRT and RCC was obtained, which was also caused by the difference in the cell morphology and tissue structure.

## 5 Conclusion

In this study, we comprehensively measured and compared the dielectric properties of human NRT and RCC from 10 Hz to 100 MHz in a strictly controlled environment (37°C, 90% humidity) within 15min after the tissue was isolated. The results showed that the dielectric properties of RCC obviously differed from NRT (the RCC conductivity was about 1.4 times as large as that of NRT); there were two characteristic frequencies (14.1 ± 1.1 kHz and 1.16 ± 0.13 MHz) for NRT while only one for RCC (0.60 ± 0.05 MHz); DCs of relative permittivity below 100 Hz and at around 14 kHz were both greater than 1. These findings further validate the feasibility of discrimination between human RCC from NRT and provide data in favor of further clinical study.

## Data Availability

The original contributions presented in the study are included in the article/supplementary material, further inquiries can be directed to the corresponding author.
